# Characterization and Optimization of Cellulose-Degrading Bacteria Isolated from Fecal Samples of *Elaphurus davidianus* Through Response Surface Methodology

**DOI:** 10.3390/microorganisms13020348

**Published:** 2025-02-06

**Authors:** Hong Wu, Chunmiao Shi, Tianyi Xu, Xinrui Dai, Dapeng Zhao

**Affiliations:** Tianjin Key Laboratory of Conservation and Utilization of Animal Diversity, College of Life Sciences, Tianjin Normal University, Tianjin 300387, China; skywuhong@tjnu.edu.cn (H.W.);

**Keywords:** metagenomic analysis, Père David’s deer, cellulose-degrading, enzyme activity, response surface analysis

## Abstract

The screening of cellulose-degrading microorganisms from herbivores and the optimization of fermentation conditions are of great significance for the utilization of cellulose resources. In this study, we initially employed a metagenomic analysis to investigate the fecal microbiota of both captive and semi-free-ranging Père David’s deer (*Elaphurus davidianus*) under varying environmental conditions. Subsequently, we isolated and cultured cellulase-degrading microorganisms from the fecal samples using Congo red medium. There was consistency in the dominant phyla and genera of gut microorganisms between the two groups, with only differences in abundance. Then, a cellulose-degrading strain identified as *Bacillus pumilus* XM was isolated after a morphological analysis and molecular identification by 16S rRNA amplicon. In addition, a series of single factor experiments and response surface analysis were conducted to determine the optimal conditions for best cellulase activity. The optimum temperature, culture time, and shaking speed for the reaction of cellulase produced by the strain *Bacillus pumilus* XM were 34 °C, 28 h, and 154 r/min, respectively. Under these conditions, the cellulase activity reached a maximum of 10.96 U/mL, which was relatively close to the predicted value of 10.975 U/mL. The results have enriched the existing bacterial resources and laid a foundation for the development of new enzymes, providing a theoretical basis for the rational utilization of cellulase from wild animal resources.

## 1. Introduction

Cellulosic biomass, which can be found in many plants, is one of the most abundant biological resources on our planet, and it can be used as fertilizer, fodder, and firewood [1,2]. Fossil fuel resources are non-renewable, and the surge in greenhouse gas emissions has resulted in environmental pollution. Conversely, cellulose resources are regarded as a potential and promising raw material that has the capacity to alleviate both the energy crisis and environmental issues [3,4]. The efficient utilization of cellulose could reduce dependence on fossil raw materials and promote sustainable development. At present, due to its complex composition and structure, the degradation and conversion of cellulosic biomass in the natural environment remain challenging, with low efficiency and numerous obstacles [5,6].

As a recyclable carbon source, the successful utilization of cellulosic materials relies on advanced technologies for cellulase production. The enzymatic hydrolysis of cellulose into glucose is the key point for the high-value conversion of cellulose because of its specificity and ecological characteristics [7]. Since cellulose is composed of glucose units linked by β-1,4-glycosidic bonds, cellulases hydrolyze these linkages with a cellulase system consisting of endoglucanase, exoglucanase, and β-glucosidase to facilitate a complete hydrolysis of cellulose into glucose [8,9].

Microbial degradation represents a biological treatment approach that contrasts with physical and chemical methods. The ability to rapidly degrade cellulose depends on the successful identification of novel strains capable of producing cellulases, and many microorganisms have been found to produce cellulases, including fungi, bacteria, and actinomycetes [10]. In general, fungi have the ability to efficiently secrete a large number of extracellular enzymes, including cellulases [11,12,13,14]. However, the culture and genetic modification of fungi were relatively difficult, and this seriously affects the promotion and application of fungi for degrading cellulose. In contrast to fungi, bacteria are commonly considered a powerful tool in cellulose production research due to the higher growth rate and expression of multi-enzyme complexes. Consequently, numerous studies have been conducted on the screening of cellulolytic bacteria. Harnvoravongchai et al. isolated and characterized the thermophilic anaerobic bacteria *Thermoanaerobacterium* sp. R63 from tropical dry deciduous forest soil with cellulose- and hemicellulose-degrading activities in northern Thailand [15]. Du et al. reported the *Massilia cellulosiltytica* sp. nov. isolated from the rhizosphere soil of rice, which contains carbohydrate enzymes to degrade cellulose [16]. He et al. isolated the cold-adapted cellulose-degrading bacteria *B. subtilis* K1, exhibiting high activity of endo-β-glucanase (24.69 U/mL) and exo-β-glucanase (1.72 U/mL) [17]. Li et al. reported the bacteria *Bacillus subtilis*, isolated from silkworm excrement, as being capable of degrading cellulose. The cellulose degradation rate was improved from 10.01% to 39.57% after treatment under the strain with high cellulase activity [18]. Unfortunately, the cellulase activity of the isolated strains was generally low, and the library of bacteria that possessed powerful cellulose activity was not sufficient. Therefore, screening for strains with elevated cellulase activity across diverse environments while optimizing their enzyme production characteristics is essential for enhancing the application potential of cellulose-degrading bacteria [19].

In recent years, numerous novel cellulose-degrading bacteria have been isolated from various environments, including soil, rotten branches, and leaves, as well as the intestines and guts of animals. The gut of herbivores with a diet of multiple foods has numerous naive cellulose-degrading bacteria, such as Koala [20], Muskoxen [21], and phytophagous insects [22]. Wild ruminants primarily consume cellulosic biomass, indicating that they may possess more efficient systems for degrading and converting cellulose.

Père David’s deer (*Elaphurus davidianus*), in the family Cervidae, belongs to the ruminant suborder and is a large herbivore that lives in swampy, muddy areas. Its main food source is the branches and leaves of Gramineae and some legumes [23]. The intestinal tract of Père David’s deer is colonized by a variety of microorganisms, which detoxify the host, provide nutrients and energy needed for growth, as well as enhance the host’s immunity. Cellulose is a major component of the diet of this herbivorous animal, and the gut microbiota of Père David’s deer plays an important role in utilizing cellulose [24]. With the development of high-throughput sequencing [25], the composition and characteristics of the gut microbiota from Père David’s deer can be obtained [26], which serves as a reference for subsequent strain isolation and screening.

Therefore, this study used high-throughput sequencing technology to analyze the differences in gut microbiota between Père David’s deer living in two different conditions and to identify functional genes/enzymes related to cellulose metabolism, clarifying the characteristics of their enzyme activity for cellulose degradation. Subsequently, traditional microbial isolation techniques using the Congo red carboxymethyl cellulose (CMC) medium were used to isolate strains from the fecal samples of Père David’s deer, followed by testing the cellulose degradation rate through DNS measurement. Then, the enzyme activity was optimized based on a response surface analysis. The findings provide a theoretical basis for comprehensive utilization of cellulosic biomass.

## 2. Materials and Methods

### 2.1. Sample Collection

In this study, fresh fecal samples of captive Pere David’s deer from Tianjin Zoo and semi-free-ranging Pere David’s deer from Tianjin Qilihai Wetland were collected. We tracked the Pere David’s deer until they defecated, and then the fecal samples were immediately collected using non-invasive sampling techniques aseptically. The fresh fecal samples were placed in sterile 5 mL EP tubes, then transported to the laboratory and stored at −80 °C for subsequent analysis. The samples were divided into the WML group (Tianjin Qilihai Wetland) and the CML group (Tianjin Zoo), according to the location of the samples. Information about the fecal samples is provided in Table S1. The research complied with the protocols established by the China Wildlife Conservation Association and the legal requirements of China without direct contact with wild animals.

### 2.2. DNA Extraction

Whole genomic DNA isolation metagenomic DNA was isolated from the fecal samples using Fecal DNA MiniPrep™ (QIAGEN, Hilden, Germany). The DNA quality was checked by visual examination of the DNA upon gel electrophoresis on 1% agarose, and DNA quantitation was performed using a NanoDrop 1000 spectrophotometer. A paired-end shotgun library was prepared according to the standard Illumina TruSeq™ DNA Sample Prep Kit protocol (Illumina, San Diego, CA, USA), and paired-end sequencing was performed on an Illumina HiSeq4000 platform (Illumina, Inc., San Diego, CA, USA) at Majorbio Bio-Pharm Technology Co., Ltd. (Shanghai, China).

### 2.3. Metagenomics Assembly and Gene Prediction

The raw FASTQ reads were quality-checked with IDBA-UD [27], and low-quality reads were removed. The open reading frames (ORFs) from each spliced contig were predicted using MetaGene [28] and the Prodial program [29]. Then, the genome assembled by the MEGAHIT tool [30] and the predicted gene sequences with a 95% sequence identity (90% coverage) were clustered using the CD-HIT software(v4.8.1) [31]. The SOAPAligner software (v2.21) was used to compare the high-quality reads of each sample with the non-redundant gene catalog, and then the abundance of genes was calculated.

A microbial diversity analysis was performed on the QIIME platform [32], and the Wilcoxon rank-sum test was used to compare the richness and evenness of the microbial community by α-diversity between two groups. A principal coordinate analysis (PCoA) based on Bray–Curtis distance was performed with the R vegan package to assess the β-diversity. Venn diagrams were constructed at the phylum/genus/species levels to compare the unique and shared gut microbiome. All open reading frames (ORFs) were functionally annotated and BLASTP against the Clusters of Orthologous Groups (COGs) of proteins and the Kyoto Encyclopedia of Genes and Genomes (KEGG) databases (http://www.genome.jp/kegg/, accessed on 6 August 2024) [33]. Additionally, the relative abundance of each key KEGG module and enzyme was calculated. Carbohydrate and active enzymes were annotated using the Carbohydrate-Active Enzymes Database (CAZy, http://www.cazy.org/, accessed on 6 August 2024) [34]. A one-way analysis of variance (ANOVA) through an FDR correction test confirmed a significant difference for metabolic pathways in the gut microbiome between the two different groups (*p* ≤ 0.05 is considered significant).

### 2.4. Isolation and Identification of Cellulose-Degrading Bacteria

Congo red staining was used in this study to isolate cellulose-degrading bacteria from fecal samples of Pere David’s deer [35]. First, fecal suspension was prepared and cultured in CMC-Na agar medium at 37 °C. After 24 h of cultivation, the agar plate was stained with a Congo red aqueous solution (2 g/L), and then colonies with the ability to decompose cellulose will form transparent circles by rinsing with 1 mol/L NaCl solution.

The ratio of the diameter of the transparent circle to the diameter of the colony (D/d) could be calculated. The higher the ratio of D/d, the stronger the ability of cellulose degradation. Those strains with high degradation potential were selected and purified to consider the growth rate and accurate calculation of cellulose-degrading capacity. After observing the morphology of the colony, a colony from the plate was inoculated into a 10 mL tube containing liquid media and incubated in a shaker incubator at 37 °C and 200 rpm. After that, the genomic DNA of the cultivated strain was extracted using a bacterial genomic DNA kit (Omega Bio-Tek, Norcross, GA, USA), according to the manufacturer’s instructions. Then, the universal bacterial primers 27F (5′-AGAGTTTGATCCTGGCTCAG-3′) and 1492R (5′-GGTTACCTTGTTACGACTT-3′) were used to apply to the 16S rRNA gene sequences. The total PCR system includes 2 × Taq Mix, DNA (50 ng/μL), primer 27F, and primer 1492R (10 μM). The resulting 16S rRNA sequences were then searched against the NCBI 16S rRNA database (http://blast.ncbi.nlm.nih.gov/, accessed on 6 August 2024) by BLAST. In order to further explain the position of the strains in the developmental evolutionary relationship, a phylogenetic analysis was performed using the Neighbor-Joining (NJ) method with bootstrap = 1000.

### 2.5. Cellulase Activity of Isolated Strain

The activity of the cellulase (CMCase) was measured by the dinitrosalicylic acid (DNS) method [36]. The bacteria XM was incubated in a 200 mL conical flask with CMC-Na (10 g/L) as the sole carbon source for 48 h. CMCase was quantified with reference to the 3,5-dinitrosalicylic acid reagent, and the standard curve was constructed by measuring the OD 520 using a 1 mg/mL glucose standard solution as the substrate. In each reaction, the crude enzyme solution was mixed with CMC-Na (pH 5.5) at 45 °C for 35 min. Then, a 3,5-dinitrosalicylic acid reagent was added for coloration. After the enzymatic reaction, the absorbance value at 520 nm was measured.

### 2.6. Response Surface Curve Analysis

Response surface methodology uses statistical and mathematical tools to develop, improve, and optimize experimental processes affected by multiple factors. In this study, the Box–Behnken design (BBD) principle was used for the experimental design, with factors affecting bacterial growth, including shaking speed (A), cultivation temperature (B), and culture time (C), set at high, medium, and low levels (1, 0, −1). The data were fitted using Design-Expert 10.0.1, and the reliability of the optimization model in the experiment was determined through a variance analysis and a significance analysis. The theoretical optimum enzyme activity was calculated, and the accuracy of the optimized culture conditions was further verified.

## 3. Results

### 3.1. Metagenomic Sequencing Results

#### 3.1.1. Host Sequences and Gene Prediction

Metagenomic sequencing of fecal samples from the captive and semi-free-ranging groups was performed on the Illumina HiSeq4000 sequencing platform. After quality control of the six samples, the final valid read data ranged from 41,252,046 to 50,828,678. After a metagenomic assembly, the data range of the assembled contigs was from 517,728 to 705,048.

The N50 value represents the total length of contigs that are equal to or greater than the N50 value, accounting for 50% of the total length. Similarly, the N90 value accounts for 90% of the total length. Generally, higher values for both N50 and N90 indicate better assembly quality. In this study, the N50 values were all above 560 bp, and the N90 values were above 330 bp. The percentage of all sample clean bases in raw bases exceeded 98%. This indicates that the genomic sequencing results were good and the gene assembly had high integrity and quality (Table S2). Subsequently, the MetaGeneMark software (v2.10) was used to predict genes for contigs larger than 500 bp, and then the CD-HIT software (v4.8.1) was used to cluster the predicted genes. The longest gene was selected as the representative sequence of each category to construct the initial non-redundant gene set. A total of 5 domains, 13 kingdoms, 177 phyla, 323 classes, 555 orders, 990 families, 2765 genera, and 10,449 species were detected from all samples in the metagenomic sequencing.

By comparing with the KEGG (Kyoto Encyclopedia of Genes and Genomes) database to obtain the KEGG functional annotation information (Pathway, Enzyme, Module, Gene) corresponding to the genes, it was found that the number of genes related to metabolism was the largest. Meanwhile, by comparing with the CAZy database (Carbohydrate-Active Enzymes Database) to obtain the functional annotation information of carbohydrate-active enzyme genes, the number of genes related to glycoside hydrolases was the largest.

#### 3.1.2. The Composition of Gut Microbiome

**Figure 1:**
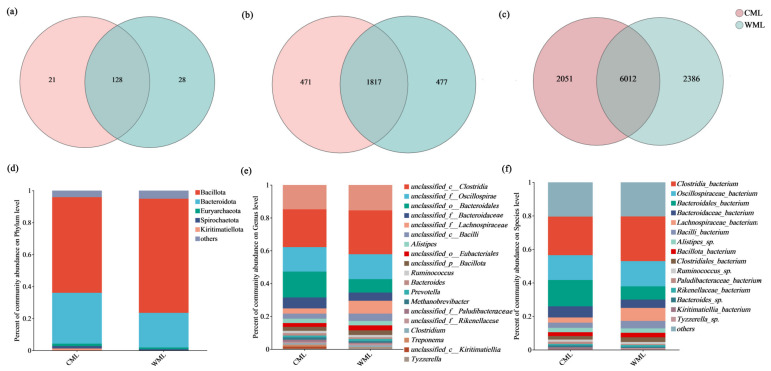
The composition of gut microbiome between CML group and WML group. The Venn diagram of species composition for the two groups at the phylum level (**a**), genus level (**b**), and species level (**c**). The composition of gut microbiome between the two groups at the phylum level (**d**), genus level (**e**), and species level (**f**).

A Venn diagram of species composition for the two groups at the phylum level, genus level, and species level is shown in Figure 1. The shared number was 128, 1817, 6012 between the captive group (CML) and the semi-captive group (WML) at phylum, genus, and species level, respectively.

At the phylum level, by comparing with the NR database, it was found that the highest proportions in the feces were Bacillota and Bacteroidota. The dominant phyla in the captive group (CML) were Bacillota (59.81%), Bacteroidota (31.79%), and Spirochaetota (1.53%). In the semi-captive group (WML), the dominant phyla were Bacillota (71.36%), Bacteroidota (21.63%), and Euryarchaeota (1.08%) (Figure 1c, Table S3). At the genus level, the dominant genera in the captive group (CML) were *unclassified_c__Clostridia* (23.02%), *unclassified_o__Bacteroidales* (15.76%), and *unclassified_f__Oscillospiraceae* (14.88%). In the semi-captive group (WML), the dominant genera were *unclassified_c__Clostridia* (26.67%), *unclassified_f__Oscillospiraceae* (15.16%), and *unclassified_o__Bacteroidales* (8.18%) (Figure 1d, Table S3). At the species level, the dominant species in the captive group (CML) were *Clostridia_bacterium* (23.02%), *Bacteroidales_bacterium* (15.70%), and *Oscillospiraceae_bacterium* (14.81%). In the semi-captive group (WML), the dominant species were *Clostridia_bacterium* (26.67%), *Oscillospiraceae_bacterium* (15.07%), and *Bacteroidales_bacterium* (7.88%) (Figure 1e, Table S3).

There was no significant difference for both the Ace index and Chao index, indicating that the diversity of the gut microbiome in the two groups was similar (Figure S1). The PCoA was used to evaluate the beta diversity of the fecal microbial composition (Figure S2). The contribution rates of PC1 and PC2 were 64.56% and 25.14%, respectively, based on Bray–Curtis distance, and there was no significant difference (*p* = 0.098). There was consistency in the dominant phyla and genera between the two sample groups, with only differences in abundance.

#### 3.1.3. Comparison of Differences in Metabolic Levels Between Two Groups

By comparing the differences in metabolic levels between the two groups, it is helpful to find microorganisms/functions with significant differences, which may be a key feature of environmental changes, such as captive and semi-free-range breeding, providing a potential source for our subsequent screening of degrading microorganisms.

**Figure 2:**
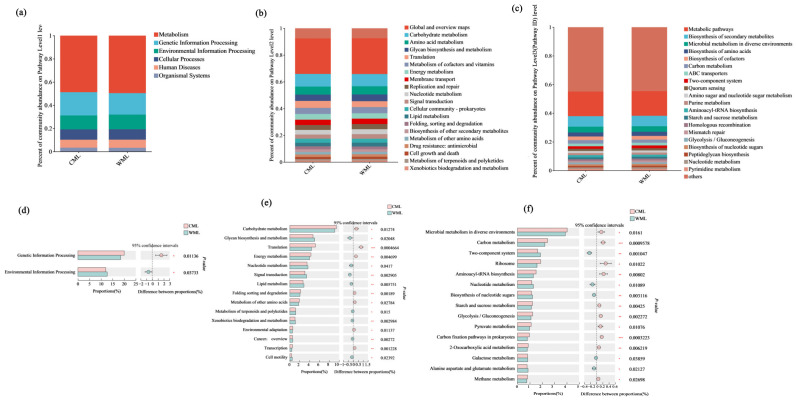
Comparison of gene abundance and differences between the two groups at the metabolic level. Metagenome-based analysis of metabolic pathway composition at KEGG level 1 (**a**), level 2 (**b**), and level 3 (**c**). Comparison of differences between the two groups at KEGG level 1 (**d**), level 2 (**e**), and level 3 (**f**). * means significant at *p* < 0.05. ** means significant at *p* < 0.01. *** means significant at *p* < 0.001.

The histogram of the KEGG pathway was calculated using the number of reads (Figure S3). Among KEGG level 1, metabolism-related pathways had the largest number of genes in both groups, followed by genetic information processing and environmental information processing. Among KEGG level 2, global and overview map pathways had the largest number of genes, followed by carbohydrate metabolism. Carbohydrate metabolism in KEGG annotations contained starch and sucrose metabolism, glycolysis/gluconeogenesis, and pyruvate metabolism. In addition, metabolic pathways (17%), biosynthesis of secondary metabolites (7%), and microbial metabolism in diverse environments (4%) were the most important pathways in KEGG level 3 (Figure 2a–c). The percent of community abundance of genetic information processing and carbohydrate metabolism in the CML group was significantly higher than that from the WML group (Figure 2d,e). At the KEGG metabolic level 3, the CML group had higher levels of carbon metabolism and glycolysis than the WML group, indicating that the genes involved in these two metabolic processes in the CML group were higher than those in the WML group (Figure 2f).

**Figure 3:**
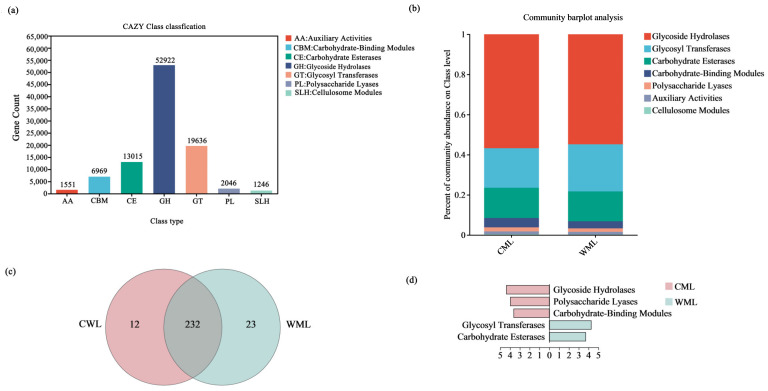
Metagenome-based analysis of carbohydrate-active enzymes. The abundance of the carbohydrate-active enzymes was calculated by the gene abundance (**a**), and the composition at the class level was compared between the CML group and the WML group (**b**). The number of genes at the GH level under the Venn diagram analysis (**c**) and the LEfSe base on CAZy (**d**).

To deepen the phylogenetic analysis, we sought to identify the potential of enzyme components that integrate into cellulosome complexes, which would be detected by the CAZyme module, including glycoside hydrolases (GHs), carbohydrate esterases, polysaccharide lyases, and carbohydrate-binding modules (CBMs) from various families (Figure 3). Furthermore, to further analyze the proportion of enzymes involved in cellulose degradation based on the CAZy database, we compared the contents of glycosyl hydrolases (GHs) and glycosyltransferases (GTs) between the two groups (Figure S4). Among the carbohydrate-active enzymes, the glycoside hydrolase family includes many cellulases. The results showed that the content of glycosyl hydrolases was higher in the CML group than in the WML group (Figure 3). However, by comparing the number of genes at the GH level under the Venn diagram analysis, the results indicated that there was a total of 232 common read numbers, while the WML group exhibited a higher number of unique reads (*n* = 23) compared with the CML group (Figure 3, Table S4). We found that the number of GH genes in the WML group was higher than that in the CML group, which indicates that there may be more genes involved in the synthesis of GHs in the fecal samples of the WML group. Therefore, we selected feces from the WML group as the source for subsequent cellulose-degrading bacteria screening.

### 3.2. Isolation and Identification of Cellulolytic Bacteria

**Figure 4:**
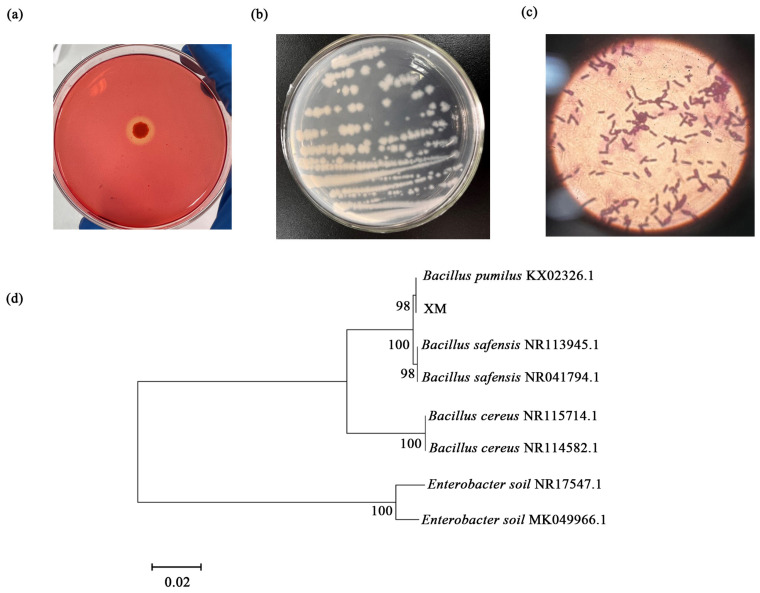
The morphological and 16S rRNA gene identification of strain XM. The bacteria XM was stained in Congo red (**a**), colony morphology (**b**), and Gram-stained morphology (**c**). The phylogenetic tree was performed using the Neighbor-Joining (NL) method (**d**).

A total of 62 strains were isolated from six fecal samples of captive Pere David’s deer from Tianjin Zoo. Based on the D/d ratio (diameter ratio between clear zone and strain) by the Congo red method, the strain that had the largest value of the D/d ratio was finally selected for further study and named XM. The colony morphological characteristics of the XM strain are shown in Figure 4. The morphological identification demonstrated that the XM strain colony is flat, opaque, white, and dull in color, rough and uneven on the surface, irregular at the edge, and waxy in texture, positive Gram staining, and microscopic rod-shaped bacteria (Figure 4a–c).

Physiological and biochemical analyses of strain XM were performed, and the results showed that the V-P test and gelatin liquefaction were both positive. The strain has the ability to utilize D-xylose, L-arabinose, D-mannitol, and starch but could not utilize citrate and propionate. The XM strain could not grow in a 7% NaCl environment but could grow under pH 5.7.

The strain XM was identified according to the 16S rRNA gene. The amplified PCR was carried out to obtain a 1500 bp fragment, which was then submitted to NCBI, and a sequence blast analysis was performed to construct a phylogenetic tree (Figure 4d). The results showed that strain XM was closely related to *Bacillus pumilus* KX023226.1. Therefore, combined with themorphological, physiological, and biochemical characterization and phylogenetic analysis, strain XM was identified as *Bacillus* sp., which suggested that the *Bacillus* might be the predominant strain possessing cellulose degradation activity from the gut sample of *Elaphurus davidianus*.

**Figure 5:**
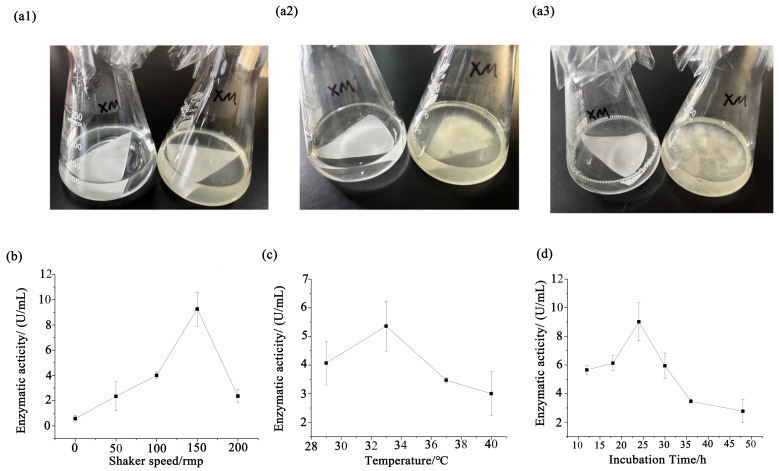
The filter paper degradation experiment and enzyme activity. Degradation effect of plastic in experimental and control groups (**a**). The filter paper degradation experiment after 2 days (**a1**), 5 days (**a2**), and 11 days (**a3**). Shown on the left is the control group, where the filter paper is placed on a blank culture medium; on the right is the experimental group, where the filter paper is placed on a medium containing bacterial solution. The enzyme activity under single factors including shaker speed (**b**), temperature (**c**), and incubation time (**d**), respectively.

After determining the basic information of the strain, we further investigated its ability to degrade cellulose. The filter paper degradation experiment was conducted, and the results can be seen in Figure 5a. The filter paper started to show minor structural disintegration after 2 days, with the medium becoming slightly turbid. The filter paper began to decay with obvious disintegration, and there were disintegrated fragments of the filter paper after 5 days. Finally, the filter paper completely disintegrated into small fragments, and there were thread-like substances in the medium with the filter paper completely disintegrated after 11 days.

Single factor tests were used to explore the best condition for enzyme activity. After 24 h of fermentation, maximum enzyme activities were achieved under 150 r/min, and if the shaking speeds became slower or higher, the enzyme activities decreased significantly (Figure 5b). The maximum enzyme activities were achieved when the culture temperature was 33 °C, and lower enzyme activities were obtained when the culture temperature was lower or higher (Figure 5c). When the cultivation time was longer or shorter than 24 h, the enzyme activities decreased (Figure 5d).

### 3.3. Statistical Optimization by Response Surface Methodology

#### 3.3.1. Box–Behnken Design

The response surface methodology (RSM) was performed to improve the activity of CMCase, a key cellulase in cellulose degradation, using the Box–Behnken design. The three key factors, including shaker, temperature, and incubation, were taken into account for enhancing the production of cellulase activity, and each variable was set up at three levels (−1, 0, +1). Based on the Box–Behnken experimental design, a three-factor, three-level response surface design was adopted (Table 1). Then, a set of 17 experiments was carried out with three variables using the Design-Expert 10.0.1 software, and the results are shown in Table 2.

By analyzing the experimental data and the quadratic equation model, the Equation is given as follows:

Yca (U/mL) = 10.61 + 0.70 A + 1.50 B + 0.79 C − 0.065 AB + 0.015 AC + 0.33 BC − 3.78 A^2^ − 2.11 B^2^ − 2.99 C^2^.

where Yca is the cellulase activity yield; and A, B, and C are the screened factors of shaker speed/rmp, temperature/°C, and incubation time/h, respectively.

The analysis was used to evaluate the accuracy and reliability of the model in predicting CMCase production under different conditions. Further, the model was determined by ANOVA to see whether there was a significant difference among the single factor and the interaction between the two factors. As shown in Table 3, it is clear that the shaking speed has a significant impact on enzyme activity. Meanwhile, the interaction analysis shows that the interactions of AB, AC, and BC are not significant (*p* > 0.1); the quadratic effects of A2, B2, and C2 are significant. Thus, there was a significant regression relationship between the cellulase activity of the XM strain and the experimental factors. The coefficient of determination (R2) of the model was 0.9858, indicating a very good fit of the model to the data, and the reliability of the predicted cellulase activity obtained by the regression equation was 98.58%. The *p* value of the misfit was 0.0522, greater than 0.05, indicating that the model’s misfit was not significant. Additionally, the model is a good representation of the data and can be used to analyze and predict the optimal CMCase activity.

#### 3.3.2. The Results of CMCase by RSM

Taking into account the interactions of the three important factors on cellulase activity of the XM strain, i.e., (A) shaker speed, (B) temperature, and (C) incubation time, they were optimized using RSM with the activity of CMCase, which is a key cellulase in cellulose degradation.

**Figure 6:**
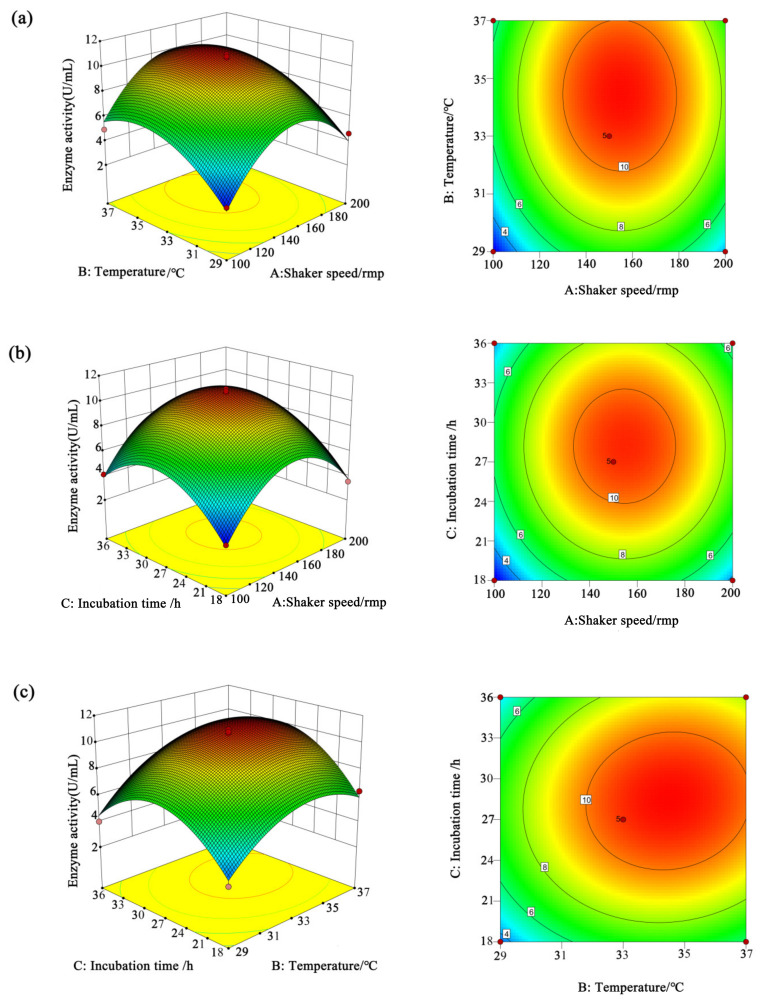
The effects of the interaction effects between the factors on CMCase activity by response surface plots. The effect of the interaction between shaker speed and culture temperature on enzyme activity (**a**). The effect of the interaction between incubation time and shaker speed on enzyme activity (**b**). The effect of the interaction between incubation time and temperature on enzyme activity (**c**).

The effects of the interactions of the factors on CMCase activity by response surfaces are shown in Figure 6. The convex shape of the response surface indicates a maximum response value and optimal variables. The contour lines are elliptical, indicating a significant interaction between the two factors.

CMCase activity first increased and then decreased with increasing the shaking speed or culture temperature. Maximum CMCase activity can be achieved at shaking speeds of 120–160 r/min and culture temperatures of 31–37 °C (Figure 6a). Similarly, the cellulase activity increases with the increase in both the shaking speed (100–155 r/min) and the cultivation time (18–28.5 h), showing a positive correlation. As shown in Figure 6b, the maximum CMCase activity can be obtained at a culture time of 24–32 h and temperature of 24–33 °C. It can be seen from Figure 6c that when the culture temperature was lower than 34.5 °C, the CMCase activity was positively correlated with the culture temperature, and the interaction between the temperature and the cultivation time had a significant effect on CMCase activity. Maximum CMCase activity can be achieved at a temperature of 29–34.5 °C and a cultivation time of 18–28.5 h. Then, Design-Expert 10.0.3 was used to further determine the optimal conditions of shaker speed, temperature, and incubation time for maximum CMCase activity. The results showed that the maximum cellulase activity of 10.975 U/mL was obtained under 154.529 r/min, 34.463 °C, and 28.368 h, respectively.

To verify the accuracy and feasibility of the design results by the response surface method, the optimal conditions obtained were used to validate the liquid fermentation of the XM strain (*Bacillus pumilus*). Under fermentation conditions where the shaking speed was set to 154 r/min, the temperature to 34 °C, and the cultivation time to 28 h, the final cellulase activity was 10.96 U/mL, which was relatively close to the predicted value of 10.975 U/mL, indicating that the established model can accurately reflect the effects on CMCase activity. Therefore, it is feasible to use the optimal factor values obtained to produce maximum cellulase by the XM strain (*Bacillus pumilus*) using the response surface method.

## 4. Discussion

Cellulose, the most abundant organic polymer on Earth, plays a pivotal role in the utilization of lignocellulosic biomass as renewable energy and raw materials. Bacterial microbial degradation is crucial in the degradation of cellulose, and the isolation of these bacteria constitutes a crucial step in elucidating the potential of diverse strains for cellulose degradation. Therefore, the continuous screening and selection of cellulose-degrading microorganisms is of great significance. According to previous reports, many microorganisms capable of degrading cellulose have been isolated from different microecological environments such as soil, compost, and anaerobic sludge; for example, Mokale Kognou et al. isolated six cellulose-degrading bacteria from mixture soil samples collected at Kingfisher Lake and the University of Manitoba campus [37]. Yadav et al. isolated the cellulolytic bacteria *Paenibacillus lautus*, strain BHU3, from a locally available landfill site [38]. In addition to these natural environments, many researchers have shifted attention toward animals, including insects and herbivorous mammals. Tang et al. isolated a cellulolytic bacterium named *Serratia marcescens* LY1 from the gut of *C. buqueti* [39]. Anand et al. isolated and characterized the bacteria that degrade cellulose from the gut of *Bombyx mori* [40]. Kumawat et al. isolated anaerobic bacteria with fiber degradation potential from feces of *Boselaphus tragocamelus* [41]. These microorganisms produce a variety of industrially valuable cellulase enzymes based on different cellulose degradation mechanisms, resulting in huge economic value. The gut microbiome from herbivorous mammals is a potent source of fibrolytic enzymes, which can help in microbial bioconversion of lignocellulose to substantial energy production. However, few reports have been published on the isolation and cultivation of cellulose-degrading bacteria from fecal samples of herbivorous mammals. The main reason is that it is more difficult to obtain feces or intestinal tissue samples of wild animals than insects, so the research in this area is stuck in obtaining samples. So far, only the feces of elephants, pandas, yaks, etc., have been documented for the isolation of cellulose-degrading microorganisms [42,43,44,45], and there was no cellulose-degrading microorganism isolated from deer. Therefore, it is necessary to screen cellulose-degrading bacteria from cellulose-rich fecal samples from wild herbivores, like deer.

The isolation and selection of cellulose-degrading bacteria is a complex process, and traditional methods such as plate counting and liquid culture have their limitations. To overcome these challenges, the advent of high-throughput technology enables the analysis of metabolic pathways in samples to identify potential target bacteria, facilitating culturable microbial isolation.

Metagenomics involves the exploration of microbial community genomes collectively, wherein sequence assembly plays a pivotal role in analysis [46]. Metagenomic assembly genomes (MAGs) are generated through the binning and mass filtering of assembled overlapping groups exhibiting similar characteristics, potentially representing highly similar strains within microbial genomes [47]. Simultaneously, genome sequence data offer valuable metabolic information such as primary metabolism and substrate utilization, providing opportunities for optimizing culture media and growth conditions for microorganisms. For example, Liang et al. found dynamic variation of bacteria and fungi during anaerobic ruminal fermentation in vitro through a metagenomic analysis [48], and they also found the composition of bacteria and fungi, as well as carbohydrate-active enzymes involved in hydrolysis and acidogenesis during fermentation in Angus bull rumen via metagenomic sequencing [49]. These methodologies have been successfully employed to isolate and cultivate novel microorganisms from diverse environments, including ocean and gut microbiota [50]. By utilizing metagenomic data obtained from acid mine drainage biofilms, Tyson et al. effectively isolated a *Leptospirillum* strain involved in nitrogen fixation by improving medium design [51]. Pope et al., through a high-throughput sequencing analysis of herbivorous Tamarwallabies’ intestinal flora, successfully cultured the WG-1 strain of *Vibrio succinicae* [52]. Lugli et al., using metagenomic sequencing, identified specific substrates associated with presumed new species and subsequently employed these substrates in culture methods to isolate novel bifidobacterium strains from animal fecal samples [53].

In this study, we conducted the metagenomic analysis of the feces from Père David’s deer in two different environments, providing initial insights into the composition and proportions of microorganisms in the feces, with Bacteroidetes being the most dominant phylum. Previous studies revealed the occurrence of diverse combinations of gut microbial compositions and functions (metagenomics) in Père David’s deer [54]. In this study, fecal samples of Père David’s deer were collected for metagenomic sequencing, followed by assembly and comparison of predicted genes to evaluate potential cellulose-degrading biosets using the KEGG pathway and glycosylhydrolase (GH) database. It was found that the bacterial family related to carbohydrate-active enzymes was more abundant in the semi-free-ranging group than in the captive group, while the carbohydrate-active enzyme family containing the glycoside hydrolase family included cellulase, suggesting the potential ability to degrade cellulose. Based on this, the semi-free-ranging Père David’s deer feces were used as the source for subsequent fiber degradation bacteria screening.

Subsequently, culture experiments were conducted using a medium formulated with various chemical compositions containing cellulose as the sole carbon source. The results revealed the successful cultivation of six strains exhibiting distinct phenotypes, among which the strain demonstrating the highest cellulase production was chosen for subsequent tests. According to previous research, the Bacillus genus was identified as one of the cellulose-degrading microorganisms isolated from various animal feces. Therefore, the Bacillus strain screened in this study holds potential for cellulase production. Through enzyme activity detection tests, we have preliminarily measured its enzyme activity of 10.96 U/mL in our strain XM (*Bacillus pumilus*), surpassing that of many previously reported strains from fecal isolates. For instance, Yang et al. (2014) isolated a Bacillus strain that exhibited an endoglucanase activity of 3.56 U/mL from pig intestine [55]. Lugani et al. (2015) reported a CMCase activity of 3.78 U/mL in *Bacillus* sp. Y3 isolated from cattle feces [56]. Compared to those strains isolated from other animal feces or intestines, our strain XM (*Bacillus pumilus*) exhibits a higher capacity for CMCase production, which can be further enhanced through optimization.

In addition to cellulase production, other physiological characteristics of the isolated strains can also be evaluated. These include growth rate, optimal temperature range, and substrate preference. Understanding these characteristics can provide insights into the suitability of different strains for industrial applications in cellulose degradation. Once the cellulose-degrading bacteria have been isolated and characterized, they can be further optimized for cellulase production. An RSM analysis can be employed to establish a mathematical model for CMCase production and analyze the significance of temperature and other factors. This optimization can be achieved through response surface methodology (RSM), which allows for the identification of the optimal conditions for cellulase production. In order to further explore the optimum fermentation conditions of the cellulose-degrading strain XM (*Bacillus pumilus*), the response surface method was used to optimize the fermentation conditions by selecting three factors: shaking speed, temperature, and time. The results showed that the cellulase activity was the highest when the shaking speed was 154.529 r/min, the temperature was 34.463 °C, and the culture time was 28.368 h, and it was estimated to reach 10.975 U/mL. In order to facilitate the experimental operation, the optimal fermentation conditions were adjusted as follows: shaking speed of 154 r/min, temperature of 34 °C, culture time of 28 h. The final cellulase activity was 10.96 U/mL, which was close to the predicted value, indicating that the response surface model was reliable. The new strain found in this study provided a good strain resource for the development and utilization of cellulose.

## 5. Conclusions

Therefore, this study conducted the metagenomic analysis of the gut microbiome composition of Père David’s deer across diverse environments and identified *Bacillus pumilus* XM as a highly efficient bacterium for cellulose degradation. Through an RSM analysis, we determined the optimal conditions for cellulase production, achieving the highest enzyme activity of 10.96 U/mL, which laid a foundation for further exploration of cellulose-degrading bacteria and enhancement of enzyme activity.

## Figures and Tables

**Table 1:** Levels of experimental design factors for enzyme activities.

Factors	Numnber		Level	
		−1	0	1
Shaker speed/rmp	A	100	150	200
Temperature/°C	B	29	33	37
Incubation time/h	C	18	27	36

A, shaker speed; B, temperature; C, incubation time. They are the three factors in the response surface test and the independent variables.

**Table 2:** Enzyme activity experimental design and experimental results.

Number	A	B	C	Enzymatic Activity U/mL
1	0	0	0	10.32
2	−1	0	1	4.13
3	1	0	1	5.19
4	1	−1	0	4.62
5	0	−1	1	4
6	0	0	0	10.68
7	0	0	0	10.26
8	−1	−1	0	2.71
9	1	0	−1	3.54
10	0	−1	−1	3.13
11	0	0	0	10.81
12	1	1	0	6.6
13	0	0	0	10.98
14	0	1	−1	6.36
15	0	1	1	8.55
16	−1	1	0	4.95
17	−1	0	−1	2.54

A, shaker speed; B, temperature; C, incubation time. They are the three factors in the response surface test and the independent variables.

**Table 3:** Analysis of the results of enzyme activity experiments.

Source	Sum of Squares	df	Mean Square		*F* Value	*p* Value	
Model	156.43	9		17.38	53.89	<0.0001	significant
A	3.95	1		3.95	12.24	0.0100	
B	18.00	1		18.00	55.80	0.0001	
C	4.96	1		4.96	15.38	0.0057	
AB	0.017	1		0.017	0.052	0.8255	
AC	0.0009	1		0.0009	0.00279	0.9593	
BC	0.44	1		0.44	1.35	0.2833	
A^2^	60.00	1		60.00	186.02	<0.0001	
B^2^	18.83	1		18.83	58.39	0.0001	
C^2^	37.52	1		37.52	116.31	<0.0001	
Residual	2.26	7		0.32			
Lack of Fit	1.87	3		0.62	6.42	0.0522	not significant
Pure Error	0.39	4		0.097			
Cor Total	158.69	16					

A, shaker speed; B, temperature; C, incubation time. AB, interaction between shaker speed and temperature; AC, interaction between shaker speed and incubation time; BC, interaction between shaker speed and temperature; A^2^, the quadratic term of shaker speed; B^2^, the quadratic term of temperature; C^2^, the quadratic term of incubation time.

## Data Availability

Raw data used in the study were uploaded to a public repository, NCBI: Bioproject PRJNA1132597.

## Supplementary Materials

The following supporting information can be downloaded at https://www.mdpi.com/article/10.3390/microorganisms13020348/s1, Figure S1: The Ace and Chao indices between the two groups at the phylum level (a,b) and genus level (c,d); Figure S2: The PCoA of fecal microbial composition between the two groups based on Bray–Curtis; Figure S3: The histogram of the KEGG pathway in this study; Figure S4: The comparison of GH levels between the two groups; Table S1: Sample collection information; Table S2: The genomic sequencing and gene assembly of six fecal samples; Table S3: Proportion of gut microbiota between captive group and semi-free-ranging group; Table S4: WML only on Class level based on CAZy.
