# Supplementary material for: Characterization and Optimization of Cellulose-Degrading Bacteria Isolated from Fecal Samples of Elaphurus davidianus Through Response Surface Methodology
Source: Microorganisms. 2025 Feb 6;13(2):348. doi: 10.3390/microorganisms13020348 (PMC11858180; doi:10.3390/microorganisms13020348)
Supplement: Supplementary file 1 [file microorganisms-13-00348-s001.zip › microorganisms-3222711-supplementary.pdf]

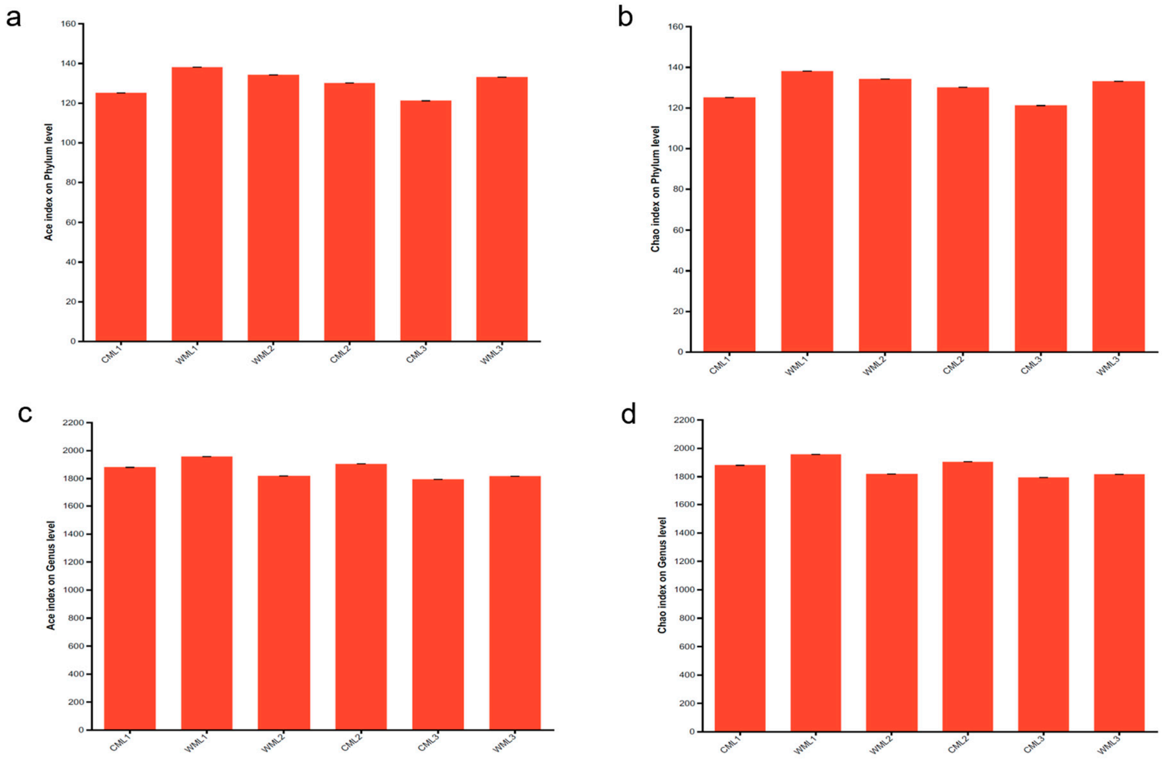

Figure S1. The Ace and Chao indexes between two groups at phylum level (a,b) and genus level (c,d)

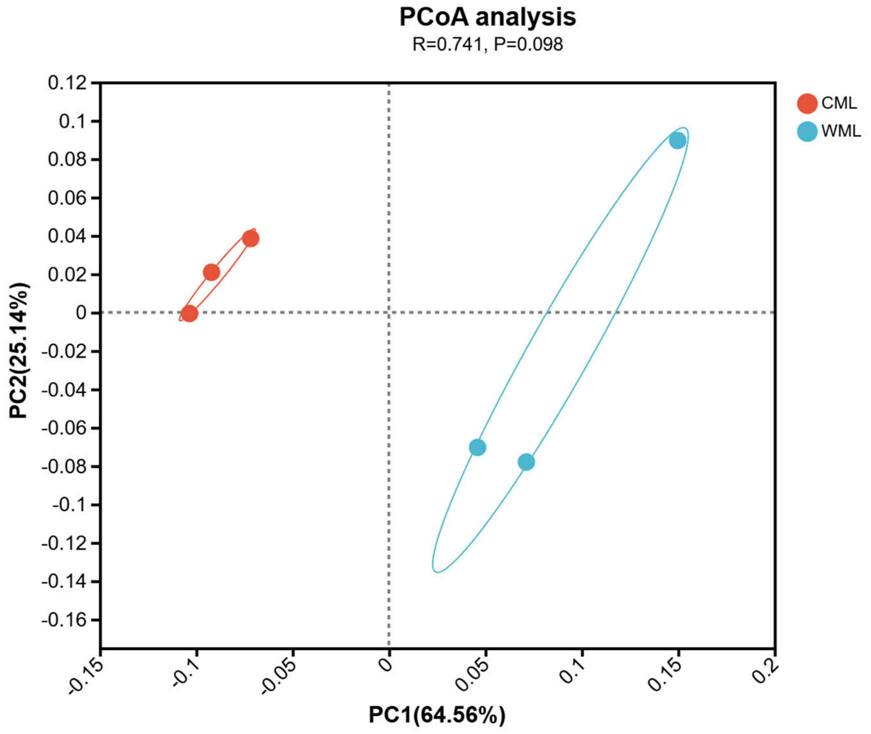

Figure S2. The PCoA of fecal microbial composition between two groups based on Bray-curtis

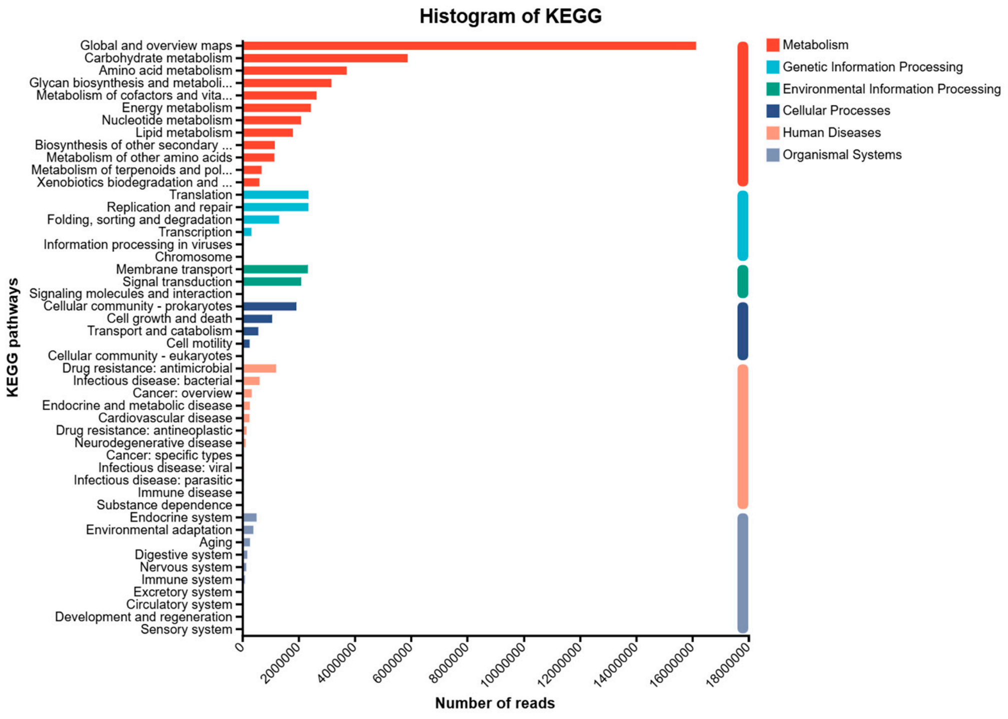

Figure S3. The histogram of KEGG pathway in this study

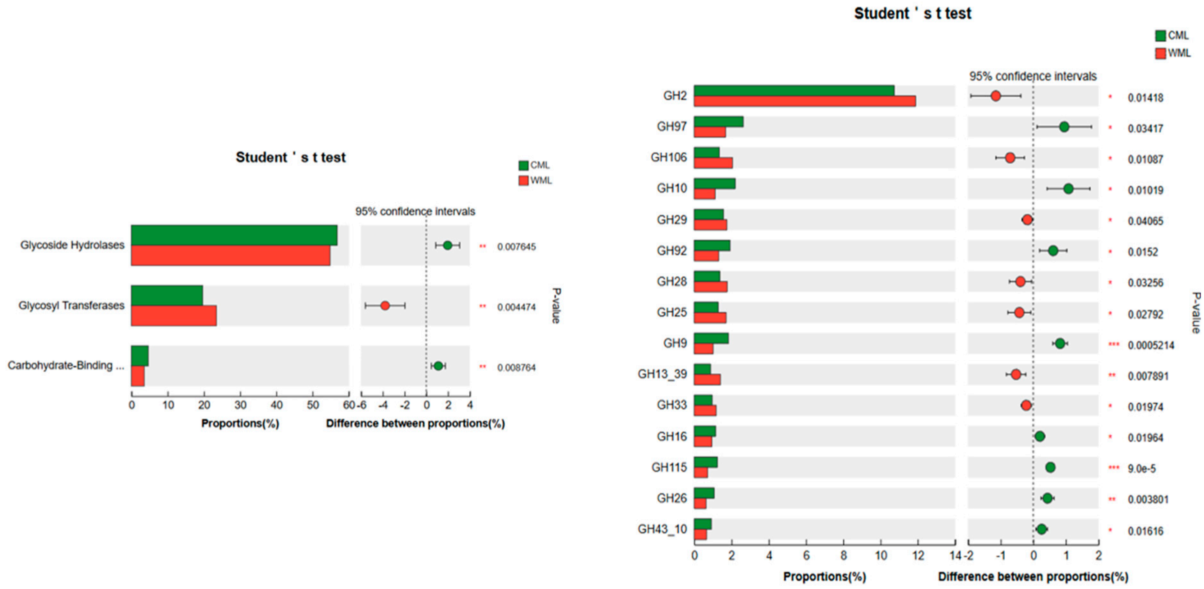

Figure S4. The comparison of GH levels between two groups

Table S1 Sample collection information

Group	Sample No.	Time of collection	Location of collection
WML	WML1	February 25, 2023	Tianjin Qilihai Wetland
	WML2	February 25, 2023	Tianjin Qilihai Wetland
	WML3	February 25, 2023	Tianjin Qilihai Wetland
CML	CML1	March 8, 2023	Tianjin Zoo
	CML2	March 8, 2023	Tianjin Zoo
	CML3	March 8, 2023	Tianjin Zoo

Table S2 The genomic sequencing and gene assembly of six fecal samples

Sample	Contigs bases(bp)	N50 (bp)	N90 (bp)	ORFs	Total Length (bp)	Average Length (bp)
CML1	404828046	577	339	859753	360132738	418.88
CML2	404392186	601	341	847468	360578853	425.48
CML3	309311778	568	338	661351	276027678	417.37
WML1	484965522	707	353	946385	432998337	457.53
WML2	361606606	723	350	695410	321989847	463.02
WML3	363500680	727	350	699277	324442413	463.97

Table S3 Proportion of gut microbiota between captive group and semi-free-ranging group

	Proportion of captive group	Proportion of the semi-free-ranging group
Phyla	Bacillota 59.81%	Bacillota 71.36%
	Bacteroidota 31.79%	Bacteroidota 21.63%
	Spirochaetota 1.53%	Euryarchaeota 1.08%
	Euryarchaeota 1.50%	Spirochaetota 0.75%
	Kiritimatiellota 1.24%	Kiritimatiellota 0.04%
Genus	*g__unclassified_c__Clostridia* 23.02%	*g__unclassified_c__Clostridia*26.67%
	g__unclassified_o__Bacteroidales 15.76%	*g__unclassified_f__Oscillospiraceae* 15.16%
	g__unclassified_f__Oscillospiraceae 14.88%	*g__unclassified_o__Bacteroidales* 8.18%
	*g__unclassified_f__Bacteroidaceae*6.66%	*g__unclassified_f__Lachnospiraceae* 7.82%
	g__unclassified_f__Lachnospiraceae 3.16%	*g__unclassified_f__Bacteroidaceae* 4.99%
	g__unclassified_c__Bacilli 3.10%	*g__unclassified_c__Bacilli* 4.47%
	g__Alistipes 2.65%	*g__unclassified_o__Eubacteriales* 3.02%
	g__unclassified_p__Bacillota 2.43%	*g__unclassified_p__Bacillota* 2.83%
	g__unclassified_o__Eubacteriales 2.30%	*g__Alistipes* 2.66%
	Bacteroides 1.47%	*g__Ruminococcus* 1.39%
Species	*s__Clostridia_bacterium* 23.02%	*s__Clostridia_bacterium* 26.67%
	s__Bacteroidales_bacterium 15.70%	*s__Oscillospiraceae_bacterium* 15.07%
	s__Oscillospiraceae_bacterium 14.81%	*s__Bacteroidales_bacterium* 7.88%
	s__Bacteroidaceae_bacterium 6.64%	*s__Lachnospiraceae_bacterium* 7.74%
	s__Lachnospiraceae_bacterium 3.11%	*s__Bacteroidaceae_bacterium* 4.97%
	s__Bacilli_bacterium 3.10%	*s__Bacilli_bacterium* 4.47%
	s__Alistipes_sp. 2.58%	*s__Clostridiales_bacterium* 2.80%
	s__Bacillota_bacterium 2.28%	*s__Bacillota_bacterium* 2.68%
	s__Clostridiales_bacterium 2.10%	*s__Alistipes_sp.* 2.62%
	s__Paludibacteraceae_bacterium: 1.33%	*s__Ruminococcus_sp.* 1.23%

Table S4 WML only on Class level based on CAZy

Name	Proportion (%)
GH5_55	43.81
GH111	5.24
GH13_24	4.76
GH13_27	4.76
GH104	4.29
GH5_32	4.29
GH43_21	3.81
GH43_14	3.81
GH13_26	3.33
GH75	2.86
others	2.86
GH5_15	2.86
GH5_14	2.38
GH62	2.38
GH5_5	1.9
GH13_12	1.9
GH5_18	1.9
GH5_41	1.43
GH13_25	1.43
